# Changes in the temperature sensitivity of SOM decomposition with grassland succession: implications for soil C sequestration

**DOI:** 10.1002/ece3.881

**Published:** 2013-11-14

**Authors:** He Nianpeng, Wang Ruomeng, Gao Yang, Dai Jingzhong, Wen Xuefa, Yu Guirui

**Affiliations:** 1 Key Laboratory of Ecosystem Network Observation and Modeling, Institute of Geographic Sciences and Natural Resources Research, Chinese Academy of SciencesBeijing, 100101, China; 2 College of Ecology and Environmental Science, Inner Mongolia Agricultural UniversityHohhot, Inner Mongolia, 010018, China

**Keywords:** Heterotrophic respiration, *Q*_10_, soil organic matter, stoichiometry, substrate, succession, warming

## Abstract

Understanding the temperature sensitivity (*Q*_10_) of soil organic matter (SOM) decomposition is important for predicting soil carbon (C) sequestration in terrestrial ecosystems under warming scenarios. Whether *Q*_10_ varies predictably with ecosystem succession and the ways in which the stoichiometry of input SOM influences *Q*_10_ remain largely unknown. We investigate these issues using a grassland succession series from free-grazing to 31-year grazing-exclusion grasslands in Inner Mongolia, and an incubation experiment performed at six temperatures (0, 5, 10, 15, 20, and 25°C) and with four substrates: control (CK), glucose (GLU), mixed grass leaf (GRA), and *Medicago falcata* leaf (MED). The results showed that basal soil respiration (20°C) and microbial biomass C (MBC) logarithmically decreased with grassland succession. *Q*_10_ decreased logarithmically from 1.43 in free-grazing grasslands to 1.22 in 31-year grazing-exclusion grasslands. *Q*_10_ increased significantly with the addition of substrates, and the *Q*_10_ levels increased with increase in N:C ratios of substrate. Moreover, accumulated C mineralization was controlled by the N:C ratio of newly input SOM and by incubation temperature. Changes in *Q*_10_ with grassland ecosystem succession are controlled by the stoichiometry of newly input SOM, MBC, and SOM quality, and the combined effects of which could partially explain the mechanisms underlying soil C sequestration in the long-term grazing-exclusion grasslands in Inner Mongolia, China. The findings highlight the effect of substrate stoichiometry on *Q*_10_ which requires further study.

## Introduction

It has become clear that the decomposition of soil organic matter (SOM) is positively correlated with temperature (Hartley and Ineson 2008; Zimmermann and Bird 2012; Zimmermann et al. 2012). The index of temperature sensitivity (*Q*_10_) has been widely used to depict the response of SOM decomposition to diurnal or seasonal temperature changes and increasingly to predict the response of soil carbon (C) sequestration in terrestrial ecosystems under warmer climate scenarios (Kirschbaum 1995; Luo et al. 2001; Knorr et al. 2005; Davidson and Janssens 2006; Conant et al. 2011).

However, the temperature sensitivity of SOM decomposition remains a highly debated topic, particularly in relation to the extent to which regional or global convergence of *Q*_10_ occurs (Zhou et al. 2009; Mahecha et al. 2010; Sierra 2012), and also regarding whether *Q*_10_ differs among soils and soil fractions (e.g., labile *vs*. recalcitrant) (Fang et al. 2005, 2006; Karhu et al. 2010a; Plante et al. 2010; Wetterstedt and Agren 2011; Tucker et al. 2013). Landscapes (e.g., grassland or forest) comprise mixed series of ecosystems in stage of succession or degradation. Understanding whether SOM decomposition and *Q*_10_ vary with ecosystem succession is important to evaluating soil C sequestration. To date, the variability of *Q*_10_ with ecosystem succession has not been extensively evaluated.

Inner Mongolian grasslands in China (about 150 million ha) have an enormous capacity to sequester atmospheric CO_2_ through rational land-use practices, especially through grazing exclusion (GE) (He et al. 2008, 2012). Soil C storage demonstrated an initial rapid increase following GE introduction and was maintained at a steady state after two decades of GE (He et al. 2008). Understanding the stability of accumulated SOM (decomposition rate and *Q*_10_) under long-term GE would be useful for accurately evaluating long-term soil C sequestration. The “carbon quality-temperature (CQT)” hypothesis predicts that the temperature sensitivity of SOM decomposition increases with biochemical recalcitrance, and this hypothesis has been tested on a variety of substrates and scales (Fierer et al. 2005; Conant et al. 2008b; Craine et al. 2010). Therefore, we assumed that *Q*_10_ should decrease with grassland succession as GE period increases because the C associated with sand and silt fractions in surface soil increase logarithmically (He et al. 2009). Moreover, the higher N:C ratio of SOM should enhance *Q*_10_ and soil C mineralization due to greater N limitation in grassland ecosystems (Billings and Ballantyne 2013).

Using a grassland succession series from free-grazing to 31-year GE, we conducted an 8-week incubation experiment using six temperatures and four substrates with differing N:C ratios. The main objectives of this study were to (1) investigate whether *Q*_10_ decreased predictably with grassland succession consistent with the CQT hypothesis, (2) determine the influence of the N:C stoichiometry of substrates on *Q*_10_ and soil C mineralization, and (3) discuss soil C sequestration mechanisms in Inner Mongolian grasslands in view of SOM decomposition.

## Material and Methods

### Study sites

The study was conducted in a typical steppe ecosystem on the Mongolian Plateau (43°33′N, 116°40′E). The area is a typical semi-arid continental climate with a mean annual temperature of 1.1°C and average annual precipitation of 345 mm (He et al. 2008). The soil is chestnut type, that is, Calcic kastanozems. The vegetation consists predominantly of grassland plants, such as *Leymus chinensis*, *Stipa grandis*, and *Cleistogenes squarrosa*.

Five grasslands, with similar vegetation and topography across in 2 km × 2 km in extent, comprised a successional series of grassland restoration, where plant communities and soil properties varied predictably after eliminating the large animal disturbance by fences (Table 1). The five plots were categorized as GE0, GE4, GE7, GE11, and GE31. Plot GE0 was subjected to long-term free-grazing and was in a slightly degraded condition in terms of the aboveground community and plant diversity. Plot GE4 was established in 2008 by fencing off a section of previously free-grazing grassland. Plots GE7, GE11, and GE31 were similarly established in 2004, 1999, and 1979, respectively (He et al. 2008). We assumed that changes in vegetation and soil properties among the five plots were mainly a result of grazing intensity and the length of GE, because the five experimental plots were floristically and topographically similar, and all were distributed in the same upper basalt platform.

**Table 1:** Changes in vegetation and soil properties with grassland succession.

Grassland type	Aboveground biomass (g m^−2^)	Soil organic carbon (g kg^−1^)	Soil total nitrogen (g kg^−1^)	Microbial biomass C (*μ*g kg^−1^)	Soil pH	Land-use history
Free-grazing grassland (GE0)	60.3 ± 20.6^a^*	13.41 ± 0.46^a^	1.43 ± 0.10^a^	47.52 ± 0.46^a^	8.17 ± 0.29^a^	Long-term free-grazing, good condition
4-year grazing exclusion (GE4)	162.3 ± 15.0^b^	15.95 ± 0.55^b^	1.60 ± 0.03^a^	42.69 ± 1.73^ab^	8.07 ± 0.11^a^	Grassland fenced since 2008, good condition
7-year grazing exclusion (GE7)	166.2 ± 13.3^b^	16.32 ± 2.06^bc^	1.64 ± 0.16^a^	38.06 ± 1.59^b^	7.92 ± 0.16^ab^	Grassland fenced since 2004, good condition
11-year grazing exclusion (GE11)	171.6 ± 9.6^b^	18.19 ± 0.49^c^	1.72 ± 0.10^a^	39.53 ± 1.89^b^	7.66 ± 0.19^b^	Grassland fenced since 1999, good condition
31-year grazing exclusion (GE31)	148.9 ± 41.3^b^	17.73 ± 2.18^bc^	1.48 ± 0.72^a^	40.02 ± 0.66^b^	7.19 ± 0.29^c^	Grassland fenced since 1979, good condition

* Data are represented as means ± 1 SD (*n* = 4). The same superscript letters within each column indicating no significant difference among grassland types at *P* < 0.05 level (ANOVA).

### Field sampling

Field sampling was conducted in July 2011. In each plot, 4 sampling quadrats (1 m × 1 m) were established at 10-m intervals along a random transect. Aboveground biomass was clipped at ground level. Soil samples in the 0–20 cm soil layer were collected from 10 points in each quadrate. In the laboratory, we manually removed roots and visible organic debris from soil samples, and then measured soil water-holding capacity (WHC, %) and soil gravimetric moisture (%). Approximately 100 g of each soil samples was air-dried for analysis of soil properties (e.g., C, N, and pH). The remaining soil was stored at 4°C.

### Laboratory incubation and analysis

The incubation experiment was conducted at six temperatures (0, 5, 10, 15, 20, and 25°C), using four substrates (control (CK), glucose (GLU), mixed grass leaf (GRA), and *Medicago falcata* leaf (MED). GRA and MED were collected from five plots and mixed evenly. The C concentration in GLU, GRA, and MED was 40.0%, 45.1%, and 44.0%, respectively, and the N concentration was 0%, 2.0%, and 5.2%, respectively. Thus, the N:C ratios of the substrates in increasing order were GLU (0) < GRA (0.043) < MED (0.117). We added 1% of the mass of the incubated soils as GLU, GRA, and MED, which approximated a 2-year input of new SOM from roots and litter in Inner Mongolian grasslands. In total, the incubation experiment comprised 480 samples, with 5 grassland types, 6 temperatures, 4 substrates, and 4 replicates for each treatment.

First, 40 g samples of fresh soils were put into incubation bottles, and the samples were adjusted to 60% WHC. Samples were incubated at 20°C and an instant 80% humidity for 4 days and then incubated at a treatment temperature (0, 5, 10, 15, 20, or 25°C) for 3 days prior to measurement of basal soil respiration. The substrates were subsequently added and mixed evenly. During the 56 days incubation experiment, soil respiration rates were measured 14 times, on days 1, 2, 3, 4, 5, 6, 7, 14, 21, 28, 35, 42, 49, and 56.

**Figure 1:**
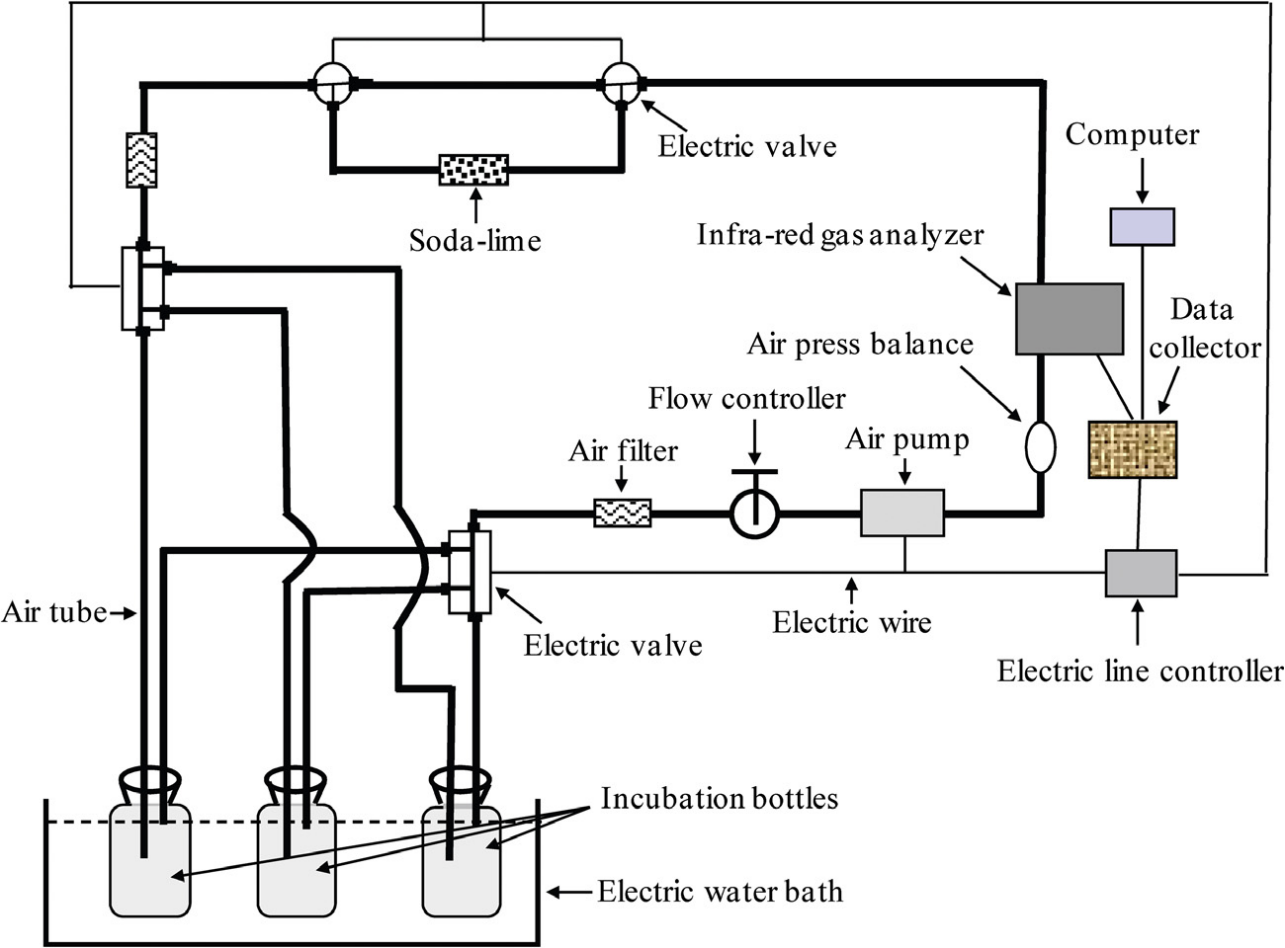
Schematic representation of the configuration of the automatic measurement system for soil microbial respiration.

An automatic system for measuring soil respiration rates was developed through modification of the continuous gas-flow system reported by Cheng et al. (1993). The system consisted of a Li-COR CO_2_ analyzer (Li-7000), an electric water bath to control incubation temperature, an air-flow controller, soda-lime equipment to control the initial CO_2_ concentration, an auto-sampler on a turn-plate, automatic transformation valves to control the sample bottle, and a data collector (Fig. 1). In practice, the system was controlled by the data collector and first automatically lowered the CO_2_ concentration by using a bypass system of soda lime and then recorded the changes in CO_2_ concentration as it steadily increased. Soil respiration rates were calculated from the slope of the CO_2_ concentration as follows:


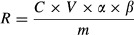


where *R* is soil respiration rate (*μ*g CO_2_ g^−1^ h^−1^); *C* is the slope of the CO_2_ concentration; *V* is the volume of the incubation bottle and gas tube; *m* is the soil weight (g); *α* is the transformation coefficient of CO_2_ mass; and *β* is the transformation coefficient of time.

Soil organic C (SOC, %) was measured using a modified Mebius method (Nelson and Sommers 1982). Soil total N (%) was measured with a modified Kjeldahl wet-digestion procedure (Gallaher et al. 1976), using a 2300 Kjeltec Analyzer Unit (FOSS Tecator, Höganäs, Sweden). Soil pH was determined using a pH meter and a slurry of soil mixed with distilled water (1:2.5). Microbial biomass C (MBC) of fresh soil samples was analyzed using the fumigation – extraction method (Vance et al. 1987).

### Calculations and statistical analysis

In this study, we selected the data from 1, 7, and 56 days incubations, respectively, to represent the instantaneous-term, short-term, and longer-term effects of the experimental treatments on soil C mineralization. Soil C mineralization at 20°C without substrate addition was used as the base soil respiration (C_min-20°C_**)**.

The temperature sensitivity (*Q*_10_) of soil respiration in the 1- and 7-day incubations was calculated using the exponential equations, because 56-day incubation should be too longer to accurately evaluate *Q*_10_ due to the respiration rates declining faster at higher temperature with faster depletion of substrate.


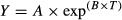



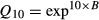


where *Y* is the soil respiration rate (*μ*gC g^−1^ h^−1^), T is the temperature (°C); *A* and *B* are the constants.

The stimulating effects (SEs) of soil respiration were then calculated to represent the sequestration capacity of grassland soils with fresh SOM input. SEs were calculated from the accumulated C mineralization under different substrates (GLU, GRA, or MED) divided by that of CK; higher values of SEs implied lower sequestration capacity.

One-way analysis of the variance (ANOVA) was used to investigate the differences in vegetation and soil properties among different grassland types. Univariate analysis of three factors (general linear model) was used to determine the effects of grassland types, incubation temperature, and substrates on soil C mineralization, *Q*_10_, and SEs. Logarithmic regression was used to identify the changing trend of *Q*_10_ and SEs with the duration of GE, MBC, and SOC. Data have been represented as means ± 1 standard deviation (*n* = 4). Differences were considered to be significant when *P* < 0.05. All analyses were conducted using SPSS statistical software (v. 13.0, SPSS, Chicago, IL, USA).

## Results

### Changes in soil properties

SOC increased significantly with increasing duration of GE (*F* = 9.18, *P* < 0.001); however, soil N content was not significantly different among grasslands. With grassland succession as a function of duration of GE, soil pH decreased from 8.17 (GE0) to 7.19 (GE31) (*F* = 15.57, *P* < 0.001) and MBC decreased logarithmically (*F* = 12.26, *P* = 0.004).

### Changes in soil C mineralization

C_min-20°C_ without the addition of external substrates differed significantly among various grasslands and decreased logarithmically with increasing duration of GE (*R*^2^ = 0.539, *P* < 0.001 for 1 day, *R*^2^ = 0.607, *P* < 0.001 for 7 days, and *R*^2^ = 0.596, *P* < 0.001 for 56 days). C_min-20°C_ was linearly correlated with MBC (*R*^2^ = 0.597, *P* < 0.001 for 1 day, *R*^2^ = 0.377, *P* = 0.004 for 7 days, and *R*^2^ = 0.382, *P* = 0.004 for 56 days).

**Figure 2:**
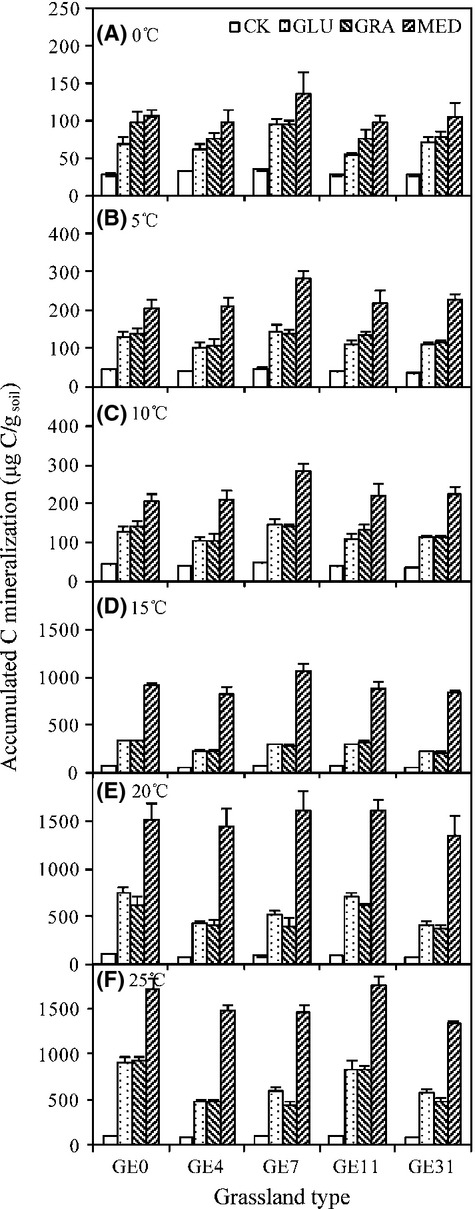
Changes in soil C mineralization with grazing-exclusion duration, substrate, and incubation temperature. CK, control; GLU, glucose; GRA, mixed grass leaf; MED, *Medicago falcate* leaf. Data were derived from 7-day incubation and represented as mean ± SD (*n* = 4).

**Figure 3:**
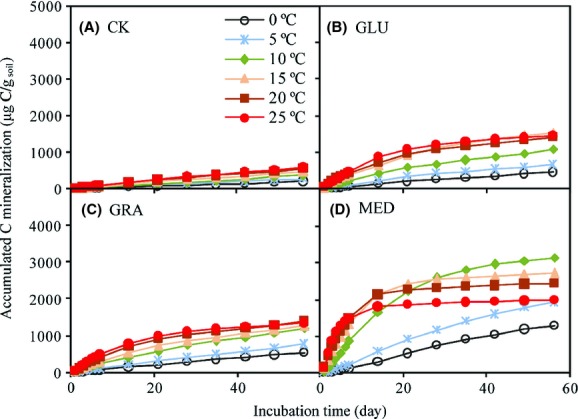
Changes in accumulated C mineralization with substrate addition (control [A], Glucose [B], Mixed grass leaf [C], and *Medicago falcata* leaf [D]) in 56-day incubation from 11-year grazing-exclusion grassland (GE11). Data were represented as mean ± SD (*n* = 4).

Grassland type, incubation temperature, and added substrates significantly influenced soil C mineralization, with notable interactive effects (Fig. 2, Table 2). Moreover, accumulated C mineralization in the CK, GLU, and GRA cases increased with temperature during the 8-week incubation in all grasslands. However, under MED conditions, accumulated C mineralization increased with temperature in the first week and then was highest at 15°C (Fig. 3).

**Table 2:** Results of univariate analysis of accumulated C mineralization (*C*_min_) according to grassland type, incubation temperature, and substrate addition.

	*C*_min-1 day_		*C*_min-7 day_		*C*_min-56 day_	
			
	*F*	*P*	*F*	*P*	*F*	*P*
Grassland type (*G*)	52.69	<0.0001	124.81	<0.0001	286.78	<0.0001
Incubation temperature (*T*)	587.31	<0.0001	2501.55	<0.0001	1253.31	<0.0001
Substrate addition (*S*)	965.76	<0.0001	6668.08	<0.0001	4701.82	<0.0001
*G* × *T*	6.75	<0.0001	23.12	<0.0001	26.57	<0.0001
*G* × *S*	6.57	<0.0001	20.86	<0.0001	60.27	<0.0001
*T* × *S*	144.25	<0.0001	555.77	<0.0001	220.02	<0.0001
*G* × *T* × *S*	1.69	0.0020	3.77	<0.0001	5.94	<0.0001

### Changes in *Q*_10_

**Figure 4:**
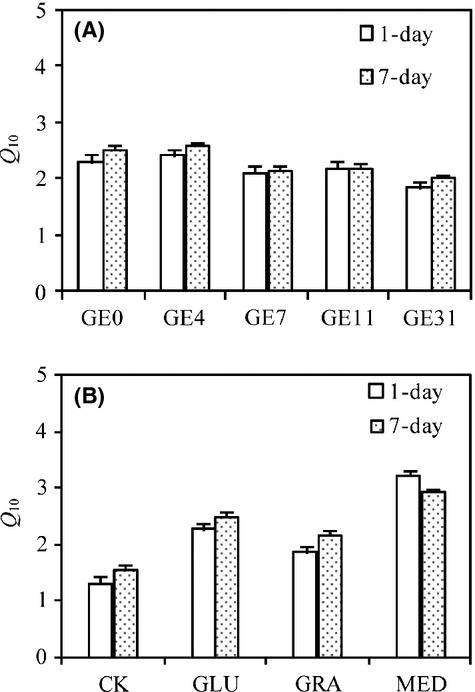
Changes in temperature sensitivity (*Q*_10_) of soil C mineralization with grassland type (A) and substrate (B). Data were represented as mean ± SD (*n* = 4).

**Figure 5:**
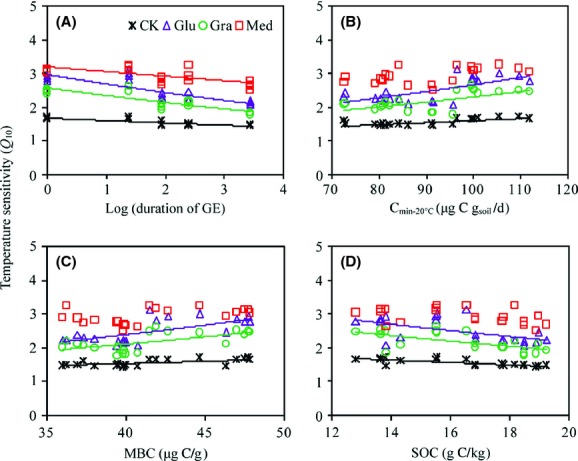
Relationship between temperature sensitivity (*Q*_10_) in 7-day incubation and duration of GE (A), C_min-20°C_ (B), MBC (C), and SOC (D).

Grassland type and added substrate had significant effects on *Q*_10_ and again showed interactions with each other (Fig. 4, Table 3). *Q*_10_ values decreased significantly with the grassland succession (*F* = 3.87, *P* = 0.011 for 1 day; *F* = 6.32, *P* < 0.001 for 7 days). *Q*_10_ values increased significantly with increasing N:C stoichiometry of substrates (*F* = 5.84, *P* = 0.001 for 1 day; *F* = 7.66, *P* < 0.001 for 7 days). *Q*_10_ in the first day were ordered as follows: CK (1.305) < GRA (1.872) < GLU (2.263) < MED (3.222). In CK, GLU, and GRA, *Q*_10_ was correlated logarithmically with C_min-20°C_, MBC, SOC, and duration of GE (Fig. 5, Table 4). However, no significant correlation was found with MED addition.

**Table 3:** Results of univariate analysis of *Q*_10_ according to grassland type and substrates.

	*Q*_10-1 day_		*Q*_10-7 day_	
		
	*F*	*P*	*F*	*P*
Grassland type (*G*)	29.37	<0.0001	136.90	<0.0001
Substrate addition (*A*)	510.44	<0.0001	958.36	<0.0001
*G* × *A*	3.65	0.0004	9.71	<0.0001

**Table 4:** Relationships of *Q*_10_ with C_min-20°C_, MBC, SOC, and duration of GE.

	C_min-20°C_ (*μ*g C g^−1^)†		MBC (*μ*g C g^−1^)‡		SOC (g kg^−1^)		Duration of GE (year)	
				
	*R*^2^	*P*	*R*^2^	*P*	*R*^2^	*P*	*R*^2^	*P*
1-day								
*Q*_10_-_CK_*	0.434§	0.002	0.510	0.001	0.413	0.003	0.693	<0.001
*Q*_10_-_GLU_	0.416	0.002	0.212	0.041	0.107	0.160	0.253	0.024
*Q*_10_-_GRA_	0.422	0.003	0.253	0.028	0.281	0.020	0.674	<0.001
*Q*_10_-_MED_	0.048	0.355	0.001	0.916	0.082	0.220	0.258	0.023
7-day								
*Q*_10_-_CK_	0.608	<0.001	0.408	0.002	0.541	<0.001	0.684	<0.001
*Q*_10_-_GLU_	0.555	<0.001	0.455	0.001	0.310	0.011	0.646	<0.001
*Q*_10_-_GRA_	0.426	0.001	0.374	0.004	0.375	0.004	0.726	<0.001
*Q*_10_-_MED_	0.161	0.080	0.168	0.072	0.144	0.099	0.471	0.001

* *Q*_10_ calculated for soil C mineralization in 1-day and 7-day incubations.

† C_min-20ºC_ is the soil respiration rate without substrate addition under 20°C after 1-day and 7-day incubation.

‡ MBC is microbial biomass C measured using the fumigation-extraction method.

§ Logarithmic equations suited most situations, identified by the minimum Akaike Information Criterion (AIC).

### Changes in SEs

**Figure 6:**
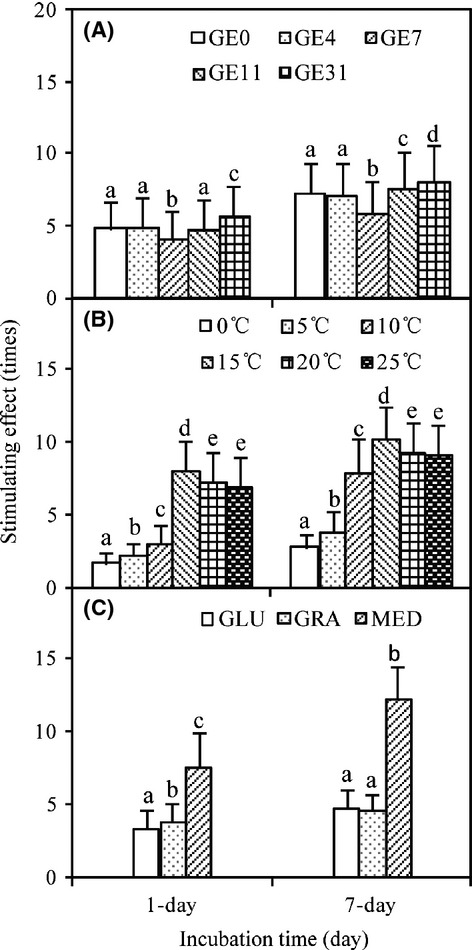
Stimulating effect of soil C mineralization according to grassland type (A), incubation temperature (B), and substrate type (C). Data with the same lowercase letters showed no significant difference at *P* = 0.05 level.

Grassland type, incubation temperature, and substrate significantly influenced SEs, with interactions among their effects (Fig. 6, Table 5). Compared with temperature and grassland type, added substrates had the strongest effect on SEs, and the effects of MED were significantly higher than those of GLU and GRA (Fig. 6).

**Table 5:** Univariate analysis of stimulating effects (SEs) according to grassland type, incubation temperature, and substrate addition.

	SE_1 day_*		SE_7 day_		SE_56 day_	
			
	*F*	*P*	*F*	*P*	*F*	*P*
Grassland type (*G*)	21.37	<0.0001	188.44	<0.0001	79.36	<0.0001
Temperature (*T*)	417.12	<0.0001	2979.44	<0.0001	216.82	<0.0001
Substrate addition (*S*)	553.31	<0.0001	4740.99	<0.0001	1042.76	<0.0001
*G* × *T*	2.97	<0.0001	94.07	<0.0001	32.68	<0.0001
*G* × *S*	5.04	<0.0001	198.83	<0.0001	79.40	<0.0001
*T* × *S*	99.79	<0.0001	1648.51	<0.0001	355.05	<0.0001
*G* × *T* × *S*	1.69	0.0082	87.02	<0.0001	37.82	<0.0001

* SE_1 day_, SE_7 day_, and SE_56 day_ represent the stimulating effect of substrates added in 1-, 7-, and 56-day incubations, respectively, which were calculated from the accumulated C mineralization for different substrates (GLU, GRA, and MED) divided by that of CK.

## Discussion

### Soil respiration with grassland succession

Soil respiration rates and MBC both decreased logarithmically with succession of grassland restoration and were significantly linearly correlated with that grazing grasslands had higher soil respiration rates, MBC, and dissolved organic C than did grazing-exclusion grasslands (Wu et al. 2012). The decrease in soil respiration with grazing exclusion mostly resulted from a decrease in MBC or from changes in microbial communities. Our results also supported the assumption that Inner Mongolian grasslands subjected to grazing exclusion have apparent C sequestration capacity as a result of depressed soil respiration (Zhou et al. 2007; He et al. 2008, 2012; Ingram et al. 2008).

These findings were not consistent with the assumption that soil respiration should increase to offset newly input SOM with grazing-exclusion grassland succession. One possible explanation is that inputs of new SOM act as a binding agents and promote the formation of soil aggregates in grazing-exclusion grasslands without large-animal trampling (Steffens et al. 2009; Wiesmeier et al. 2012; Zimmermann et al. 2012). Decreases in MBC and microbial activity and greater physical protection of SOM by increased soil aggregation (Plante et al. 2009) should be the mechanism underlying increased C sequestration in soils in these long-term grazing-exclusion grasslands in Inner Mongolia.

### Temperature sensitivity with ecosystem succession

*Q*_10_ decreased logarithmically with grassland restoration succession and was negatively correlated with the content of SOC, which is in agreement with the “C quality-temperature” hypothesis, that *Q*_10_ should be inversely related to C quality (Conant et al. 2008a; Craine et al. 2010; Xu et al. 2012). The C associated with sand and silt fractions in the surface soil increased logarithmically as the period of GE increased (He et al. 2009), which implied greater instability of SOM and lower *Q*_10_. This issue is still highly debated, although a large number of studies have focused on *Q*_10_ from various soil types, soil profiles, land-use types, and latitude gradients (Pavelka et al. 2007; Vanhala et al. 2008; Karhu et al. 2010b; Schindlbacher et al. 2010; Jenkins and Adams 2011). Zhou et al. (2009) demonstrated that the global pattern of *Q*_10_ varied from 1.40 to 2.03. Mahecha et al. (2010) found *Q*_10_ to be globally convergent on 1.40, independent of mean annual temperature, and invariant among biomes.

### Temperature sensitivity with different stoichiometry of input SOM

Stoichiometry of input SOM (N:C ratios) has a significant influence on the *Q*_10_ of SOM decomposition, which implies that the Michaelis–Menten equation 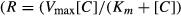 appropriately depicts the response of soil respiration after the addition of substrates because the canceling effect of *K*_m_ on the apparent *Q*_10_ is greatly reduced by substrate saturation. Gershenson et al. (2009) reported that substrate availability had a significant positive effect on *Q*_10_. Compared with GLU and GRA, MED had a significantly greater effect on *Q*_10_, which implies that substrates with higher N:C will facilitate *Q*_10_ by reducing microbial N limitation in the process of SOM decomposition. Similarly, Billings and Ballantyne (2013) demonstrated that any reduction in the C:N flow ratio resulting from temperature increase could exacerbate extant C limitation or shift microbes from N limitation to C limitation. Our findings supported the assumption of Billings and Ballantyne (2013) and further highlight the importance of stoichiometry of substrate.

In theory, a negative respiratory response to warming should be observed as microbes experience greater relative C limitation, although such a response may be mediated by shifts in function within the microbial community (Luo et al. 2001). The N:C ratios of added substrates significantly affected *Q*_10_; however, it is still not clear whether *Q*_10_ is linearly related to the N:C ratio of these substrates. Therefore, changes in the plant community and in N:C ratios of new SOM inputs should influence the stability of intrinsic SOM and soil C sequestration potential, through regulation of *Q*_10_ by altering the stoichiometry of new inputs of SOM from root exudates in Inner Mongolian grasslands (Kong et al. 2011). If this holds true, achieving increased soil C storage by sowing legumes, as confirmed by many studies, should be re-evaluated under warming scenarios.

### SEs with grassland succession and stoichiometry of input SOM

Grassland type, incubation temperature, and added substrates influenced SEs of CO_2_ emission after the addition of substrates. SEs were higher in GE0 and GE31, but lower in GE4, GE7, and GE11, which implied that the soils in GE0 and GE31 had lower C sequestration for a given input of SOM. This finding provides one of underlying mechanisms for an initial rapid increase in soil C storage following GE introduction followed by relatively stable levels after 2 decades of GE (He et al. 2008; Wu et al. 2008). Temperature influences the magnitude and duration of SEs after substrates are added. SEs increased with increasing temperature in the first stage and then reached a maximum at 15°C for GLU, GRA, and MED addition. Increase in the higher N:C ratio of the substrates added led to increased SEs in the first stage, but these were of shorter duration. The possible explanations for this are as follows: (1) addition of higher N:C substrates temporarily improves SOM quality and accelerates soil respiration, but this then leads to microbial N limitation when labile SOM is depleted (Billings and Ballantyne 2013), (2) temperature affects soil microbial enzyme activity, and 15°C should be the optimum temperature at which limitation of SOM quality after substrate addition does not occur.

In conclusion, the *Q*_10_ of SOM decomposition decreased logarithmically with grassland restoration succession and was negatively correlated with SOC content. The stoichiometry (N:C) of new SOM input had an apparent influence on soil C mineralization and temperature sensitivity, and higher N:C ratios led to higher *Q*_10_ levels and stronger soil C mineralization. Our findings imply that changes in the plant community and in the N:C ratios of new SOM input from litter, root, and roots exudates would influence the stability of intrinsic SOM and soil C sequestration potential through regulation of *Q*_10_. Overall, changes in *Q*_10_ with ecosystem succession are controlled by MBC, SOM quality, and the stoichiometry of new input SOM; these should be the underlying ecosystem-level mechanisms for soil C sequestration in grazing-exclusion grasslands.

## References

[b1] Billings SA, Ballantyne F (2013). How interactions between microbial resource demands, soil organic matter stoichiometry, and substrate reactivity determine the direction and magnitude of soil respiratory responses to warming. Glob. Change Biol.

[b100] Cheng WX, Virginia RA (1993). Measurement of microbial biomass in Arctic tundra soils using fumigation extraction and substrate-induced respiration procedures. Soil Biol. Biochem.

[b2] Conant RT, Drijber RA, Haddix ML, Parton WJ, Paul EA, Plante AF (2008a). Sensitivity of organic matter decomposition to warming varies with its quality. Glob. Change Biol.

[b3] Conant RT, Steinweg JM, Haddix ML, Paul EA, Plante AF, Six J (2008b). Experimental warming shows that decomposition temperature sensitivity increases with soil organic matter recalcitrance. Ecology.

[b4] Conant RT, Ryan MG, Agren GI, Birge HE, Davidson EA, Eliasson PE (2011). Temperature and soil organic matter decomposition rates - synthesis of current knowledge and a way forward. Glob. Change Biol.

[b5] Craine J, Spurr R, Mclauchlan K, Fierer N (2010). Landscape-level variation in temperature sensitivity of soil organic carbon decomposition. Soil Biol. Biochem.

[b6] Davidson EA, Janssens IA (2006). Temperature sensitivity of soil carbon decomposition and feedbacks to climate change. Nature.

[b7] Fang CM, Smith P, Moncrieff JB, Smith JU (2005). Similar response of labile and resistant soil organic matter pools to changes in temperature. Nature.

[b8] Fang C, Smith P, Smith JU (2006). Is resistant soil organic matter more sensitive to temperature than the labile organic matter?. Biogeosciences.

[b9] Fierer N, Craine JM, McLauchlan K, Schimel JP (2005). Litter quality and the temperature sensitivity of decomposition. Ecology.

[b10] Gallaher RN, Weldon CO, Boswell FC (1976). A semi-automated procedure for total nitrogen in plant and soil samples. Soil Sci. Soc. Am. J.

[b11] Gershenson A, Bader NE, Cheng WX (2009). Effects of substrate availability on the temperature sensitivity of soil organic matter decomposition. Glob. Change Biol.

[b12] Hartley IP, Ineson P (2008). Substrate quality and the temperature sensitivity of soil organic matter decomposition. Soil Biol. Biochem.

[b13] He NP, Yu Q, Wu L, Wang YS, Han XG (2008). Carbon and nitrogen store and storage potential as affected by land-use in a *Leymus chinensis* grassland of northern China. Soil Biol. Biochem.

[b14] He NP, Wu L, Wang YS, Han XG (2009). Changes in carbon and nitrogen in soil particle-size fractions along a grassland restoration chronosequence in northern China. Geoderma.

[b15] He NP, Zhang YH, Dai JZ, Han XG, Baoyin TGT, Yu GR (2012). Land-use impact on soil carbon and nitrogen sequestration in typical steppe ecosystems, Inner Mongolia. J. Geog. Sci.

[b16] Ingram LJ, Stahl PD, Schuman GE, Buyer JS, Vance GF, Ganjegumte GK (2008). Grazing impacts on soil carbon and microbial communities in a mixed-grass ecosystem. Soil Sci. Soc. Am. J.

[b17] Jenkins ME, Adams MA (2011). Respiratory quotients and *Q*_10_ of soil respiration in sub-alpine Australia reflect influences of vegetation types. Soil Biol. Biochem.

[b18] Karhu K, Fritze H, Hamalainen K, Vanhala P, Jungner H, Oinonen M (2010a). Temperature sensitivity of soil carbon fractions in boreal forest soil. Ecology.

[b19] Karhu K, Fritze H, Tuomi M, Vanhala P, Spetz P, Kitunen V (2010b). Temperature sensitivity of organic matter decomposition in two boreal forest soil profiles. Soil Biol. Biochem.

[b20] Kirschbaum MUF (1995). The temperature-dependence of soil organic-matter decomposition, and the effect of global warming on soil organic-C storage. Soil Biol. Biochem.

[b21] Knorr W, Prentice IC, House JI, Holland EA (2005). Long-term sensitivity of soil carbon turnover to warming. Nature.

[b22] Kong DL, Wu HF, Zeng H, Lü XT, Simmons M, Wang M (2011). Plant functional group removal alters root biomass and nutrient cycling in a typical steppe in Inner Mongolia, China. Plant Soil.

[b23] Luo YQ, Wan SQ, Hui DF, Wallace LL (2001). Acclimatization of soil respiration to warming in a tall grass prairie. Nature.

[b24] Mahecha MD, Reichstein M, Carvalhais N, Lasslop G, Lange H, Seneviratne SI (2010). Global convergence in the temperature sensitivity of respiration at ecosystem level. Science.

[b25] Nelson DW, Sommers LE, Page AL, Miller RH, Keeney DR (1982). Total carbon, organic carbon, and organic matter. Methods of soil analysis.

[b26] Pavelka M, Acosta M, Marek MV, Kutsch W, Janous D (2007). Dependence of the *Q*_10_ values on the depth of the soil temperature measuring point. Plant Soil.

[b27] Plante AF, Six J, Paul EA, Conant RT (2009). Does physical protection of soil organic matter attenuate temperature sensitivity?. Soil Sci. Soc. Am. J.

[b28] Plante AF, Conant RT, Carlson J, Greenwood R, Shulman JM, Haddix ML (2010). Decomposition temperature sensitivity of isolated soil organic matter fractions. Soil Biol. Biochem.

[b29] Schindlbacher A, Diaz-Pines C, De Gonzalo E, Gorria P, Matthews B, Inclan R (2010). Temperature sensitivity of forest soil organic matter decomposition along two elevation gradients. J. Geophys. Res. Biogeosci.

[b30] Sierra CA (2012). Temperature sensitivity of organic matter decomposition in the Arrhenius equation: some theoretical considerations. Biogeochemistry.

[b32] Steffens M, Kolbl A, Kögel-Knabner I (2009). Alteration of soil organic matter pools and aggregation in semi-arid steppe topsoils as driven by organic matter input. Eur. J. Soil Sci.

[b33] Tucker CL, Bell J, Pendall E, Ogle K (2013). Does declining carbon-use efficiency explain thermal acclimation of soil respiration with warming?. Glob. Change Biol.

[b34] Vance ED, Brookes PC, Jenkinson DS (1987). An extraction method for measuring soil microbial biomass C. Soil Biol. Biochem.

[b35] Vanhala P, Karhu K, Tuomi M, Bjorklof K, Fritze H, Liski J (2008). Temperature sensitivity of soil organic matter decomposition in southern and northern areas of the boreal forest zone. Soil Biol. Biochem.

[b36] Wetterstedt JM, Agren GI (2011). Quality or decomposer efficiency - which is most important in the temperature response of litter decomposition? A modelling study using the GLUE methodology. Biogeosciences.

[b37] Wiesmeier M, Steffens M, Mueller CW, Kolbl A, Reszkowska A, Peth S (2012). Aggregate stability and physical protection of soil organic carbon in semi-arid steppe soils. Eur. J. Soil Sci.

[b38] Wu L, He NP, Wang YS, Han XG (2008). Storage and dynamics of carbon and nitrogen in soil following grazing exclusion in *Leymus chinensis* grasslands of northern China. J. Environ. Qual.

[b39] Wu HH, Wiesmeier M, Yu Q, Steffens M, Han XG, Kogel-Knabner I (2012). Labile organic C and N mineralization of soil aggregate size classes in semiarid grasslands as affected by grazing management. Biol. Fertil. Soils.

[b40] Xu X, Luo YQ, Zhou JZ (2012). Carbon quality and the temperature sensitivity of soil organic carbon decomposition in a tallgrass prairie. Soil Biol. Biochem.

[b41] Zhou ZY, Sun OJ, Huang JH, Li LH, Liu P, Han XG (2007). Soil carbon and nitrogen stores and storage potential as affected by land-use in an agro-pastoral ecotone of northern China. Biogeochemistry.

[b42] Zhou T, Shi PJ, Hui DF, Luo YQ (2009). Global pattern of temperature sensitivity of soil heterotrophic respiration (*Q*_10_) and its implications for carbon-climate feedback. J. Geophys. Res. Biogeosci.

[b43] Zimmermann M, Bird MI (2012). Temperature sensitivity of tropical forest soil respiration increase along an altitudinal gradient with ongoing decomposition. Geoderma.

[b44] Zimmermann M, Leifeld J, Conen F, Bird MI, Meir P (2012). Can composition and physical protection of soil organic matter explain soil respiration temperature sensitivity?. Biogeochemistry.

